# Automated Kellgren–Lawrence grading of knee osteoarthritis using a multi-scale attention-based deep learning framework

**DOI:** 10.3389/fmed.2026.1809768

**Published:** 2026-06-15

**Authors:** Henghui Zhang, Chui Kong, Hanwen Chang, Yaokai Gan

**Affiliations:** 1 Shanghai Key Laboratory of Orthopaedic Implants, Department of Orthopaedic Surgery, Shanghai Jiao Tong University School of Medicine, School of Biomedical Engineering, Shanghai Ninth People’s Hospital, Shanghai Jiao Tong University, Shanghai, China; 2 School of Information Science and Technology, Fudan University, Shanghai, China

**Keywords:** accuracy, algorithm, deep learning, framework, knee osteoarthritis

## Abstract

**Background:**

Accurate radiographic grading of knee osteoarthritis (KOA) using the Kellgren–Lawrence (KL) system is essential for clinical decision-making but suffers from subjective interpretation and inter-observer variability. Deep learning approaches have shown promise, yet existing models often rely on single-scale features, lack effective attention mechanisms, and generalize poorly to external datasets.

**Methods:**

We developed a novel multi-scale attention-guided deep learning framework that synergistically integrates three key components: (1) a Feature Pyramid Network (FPN) for multi-scale feature fusion across different spatial resolutions; (2) a dual-attention mechanism combining a Squeeze-and-Excitation (SE) module (channel-wise recalibration) and a Self-Attention module (long-range spatial dependency modeling); and (3) knowledge distillation to enhance generalization. A total of 5,000 radiographs from the OSAIL KL dataset were used for model development (3,500 training, 1,500 internal validation), with external validation on an independent dataset of 2,000 radiographs. Performance was compared against ResNet50, ResNet18, MobileNet V2, and DenseNet using 5-fold cross-validation.

**Results:**

On internal validation, our model achieved superior performance across all metrics (F1: 0.726, precision: 0.740, MCC: 0.620, accuracy: 0.726), outperforming all baseline architectures. On external validation, the model maintained robust generalization (F1: 0.656, precision: 0.683, MCC: 0.564, accuracy: 0.685), with only a 4.04% drop in accuracy—demonstrating clinical reliability. Misclassifications were predominantly confined to adjacent KL grades, with no extreme errors (e.g., Grade 0 vs. Grade 4). Gradient-weighted Class Activation Mapping (Grad-CAM) heatmaps revealed that the model’s attention aligns with clinically relevant regions (joint space, osteophyte sites), enhancing interpretability.

**Conclusion:**

The proposed multi-scale attention-guided framework enables reliable, automated KL grading of KOA from radiographs with strong generalization across independent datasets. By integrating FPN, dual-attention mechanisms, and knowledge distillation, this approach addresses key limitations of prior models and may support standardized, objective, and interpretable clinical assessment of KOA severity.

## 1 Introduction

Osteoarthritis (OA) is a common degenerative joint disease affecting a large and growing global population, driven by factors like population aging and rising obesity rates (1). Characterized by cartilage damage, sub-bone remodeling, osteophyte formation, joint capsule changes, and synovial inflammation (2). OA poses a significant health burden. According to the 2021 Global Burden of Disease Study, around 607 million people worldwide suffer from OA, with knee osteoarthritis (KOA) being the predominant subtype (3). KOA causes severe pain and functional impairment, greatly impacting patients’ quality of life (4, 5).

In clinical practice, the severity of KOA, along with the nature of bone deformities and intra-articular pathologies, is crucial for guiding treatment decisions (6). For example, mild to moderate KOA may be managed with analgesics and non-steroidal anti-inflammatory drugs (NSAIDs), while severe cases might require more potent medications or combination therapies (7). Surgical interventions such as total knee arthroplasty (TKA) may be recommended for significant joint damage, deformity correction, or severe pain unresponsive to conservative treatments (8). The specific bone deformities and pathologies also influence the choice of surgical technique (9). Radiographs are essential for preoperative planning and surgical execution, providing detailed joint structure information (10).

With the rapid development of artificial intelligence (AI), deep learning (DL), a subset of AI, has shown great potential in medical image analysis (11). Unlike traditional machine learning (ML) that often requires extensive feature extraction before training, DL algorithms can autonomously learn features from data, improving analysis at every stage (12). However, DL is an auxiliary tool for clinicians, aiming to enhance decision-making accuracy and efficiency (13).

In orthopedics, DL can assist radiologists and orthopedic surgeons in interpreting medical images automatically. It can improve diagnostic accuracy and speed, reduce human errors caused by fatigue and inexperience, and alleviate medical professionals’ workload (14). DL methods trained on senior specialists’ expertise can transfer knowledge to smaller institutions, improving care access in areas with scarce experts (15). DL has been successful in various orthopedic domains, including fracture detection, bone tumor diagnosis, and KOA grading (16, 17).

Several studies have focused on AI in KOA. Pongsakonpruttikul et al. conducted a cross-sectional diagnostic study on AI-assisted radiographic detection and classification of KOA severity (18). Cigdem et al. provided a comprehensive review of AI in KOA for 2022 (19). Brejnebol validated an AI tool for radiographic KOA severity classification (20), and Abdullah et al. explored automatic detection and classification of KOA using DL (21). Some studies have also used open-source Kaggle datasets for KOA-related research (22).

However, these studies have limitations. Many fail to comprehensively integrate multi-scale spatial information, relying instead on single-scale feature extraction that limits accurate identification of early-stage KOA. Existing models like ResNet50, ResNet18, and DenseNet often overfit on smaller datasets and may not effectively capture multi-scale spatial features (23, 24). ResNet50’s deep architecture requires substantial computational resources and may struggle to distinguish similar severity levels; DenseNet’s dense connections can introduce redundant information, increasing computational burden and potentially interfering with focus on key features. Moreover, performance is highly dependent on training data quality and diversity, limiting practical application in real-world clinical scenarios.

Furthermore, while there is growing research on AI and ML in musculoskeletal imaging, specific algorithms like Support Vector Machines (SVMs), Random Forests, and Convolutional Neural Networks (CNNs) have been used. SVMs have been used for classifying musculoskeletal abnormalities based on manually extracted image features (25). Random Forests have shown promise in bone age assessment and fracture detection, handling high-dimensional data and providing feature importance scores (26). CNNs have been widely adopted for automatic feature extraction and classification in musculoskeletal images, demonstrating excellent performance in detecting OA in knee X-rays (27). However, there is still limited research on using AI for staging KOA, and existing literature has not fully addressed the need for a model that can integrates diverse spatial information with robust generalization.

To address these limitations, this study proposes a novel DL framework that integrates a Feature Pyramid Network (FPN) for multi-scale feature fusion, spatial attention mechanisms (SE Module and Self-Attention) to highlight relevant regions, and knowledge distillation to improve generalization. As summarized in Table 1, the novelty of our framework lies not merely in the use of these individual components, but in their synergistic and tailored integration for automated KOA grading. Unlike standard FPN-based models that lack explicit spatial guidance, our framework employs a dual-attention mechanism (SE + Self-Attention) operating on the multi-scale features. The SE Module recalibrates channel-wise feature responses, emphasizing informative channels, while the Self-Attention captures long-range spatial dependencies, allowing the model to correlate subtle local signs (e.g., early osteophytes) with broader joint structures (e.g., global alignment). Furthermore, in contrast to conventional attention-based models that typically operate on single-scale features, our approach applies these attention mechanisms within a multi-scale FPN hierarchy. This specific design is clinically motivated for KOA grading, which inherently requires simultaneous assessment of both fine-grained details (e.g., marginal osteophytes) and large-scale structural changes.

**TABLE 1:** Comparison of key architectural components and novelties of the proposed framework against existing deep learning models for medical image analysis.

Feature	ResNet based models (e.g., ResNet50/18) (22, 24)	DenseNet (25, 37)	MobileNet V2 (35)	Standard FPN-based models	Standard attention-based models (e.g., SE-Net, ViT) (32, 33)	Our proposed framework
Backbone	ResNet (50/18)	DenseNet	MobileNet V2	ResNet/EfficientNet	Various (ResNet, ViT)	EfficientNet
Multi-scale feature extraction	Single-scale (from final layer)	Single-scale (dense connections within blocks)	Single-scale	Yes (FPN)	Not primary focus	Yes (FPN)
Attention mechanism	None inherent	None inherent	None inherent	None or basic (e.g., SE)	Yes (e.g., SE, self-attention)	Synergistic integration of FPN + SE Module + Self-Attention
Key design purpose/novelty	General feature extraction	Feature reuse and gradient flow	Efficiency for mobile/edge devices	Multi-scale feature fusion	Global/local feature weighting	Hierarchical multi-scale attention: FPN extracts features at multiple scales; SE and Self-Attention jointly refine them, focusing on both channel-wise importance and long-range spatial dependencies.
Application to KOA grading/limitation	Standard baseline. may miss subtle, multi-scale signs (e.g., early osteophytes).	Prone to overfitting on smaller medical datasets. may focus on redundant features.	Lower accuracy for nuanced grading where fine texture details are crucial.	Lacks explicit spatial guidance to focus on the most pathologically relevant features across scales.	–	–

### 1.1 Key contributions

Contribution 1: A novel multi-scale attention-guided framework that synergistically integrates FPN, SE module, and Self-Attention, tailored for KL grading.

Contribution 2: Demonstration of superior and robust performance against widely used baselines, with minimal “dangerous” cross-grade errors.

Contribution 3: Enhanced generalization via knowledge distillation, validated by an external accuracy drop of only 4.04%.

Contribution 4: Clinical interpretability through Grad-CAM heatmaps that align with clinically relevant regions.

## 2 Materials and methods

### 2.1 Ethical statement

All data are from public databases and do not involve ethical approval. This article does not contain any studies with human participants conducted by the authors. All data were obtained from public databases and did not involve patient consent.

### 2.2 Overall workflow

**FIGURE 1:**
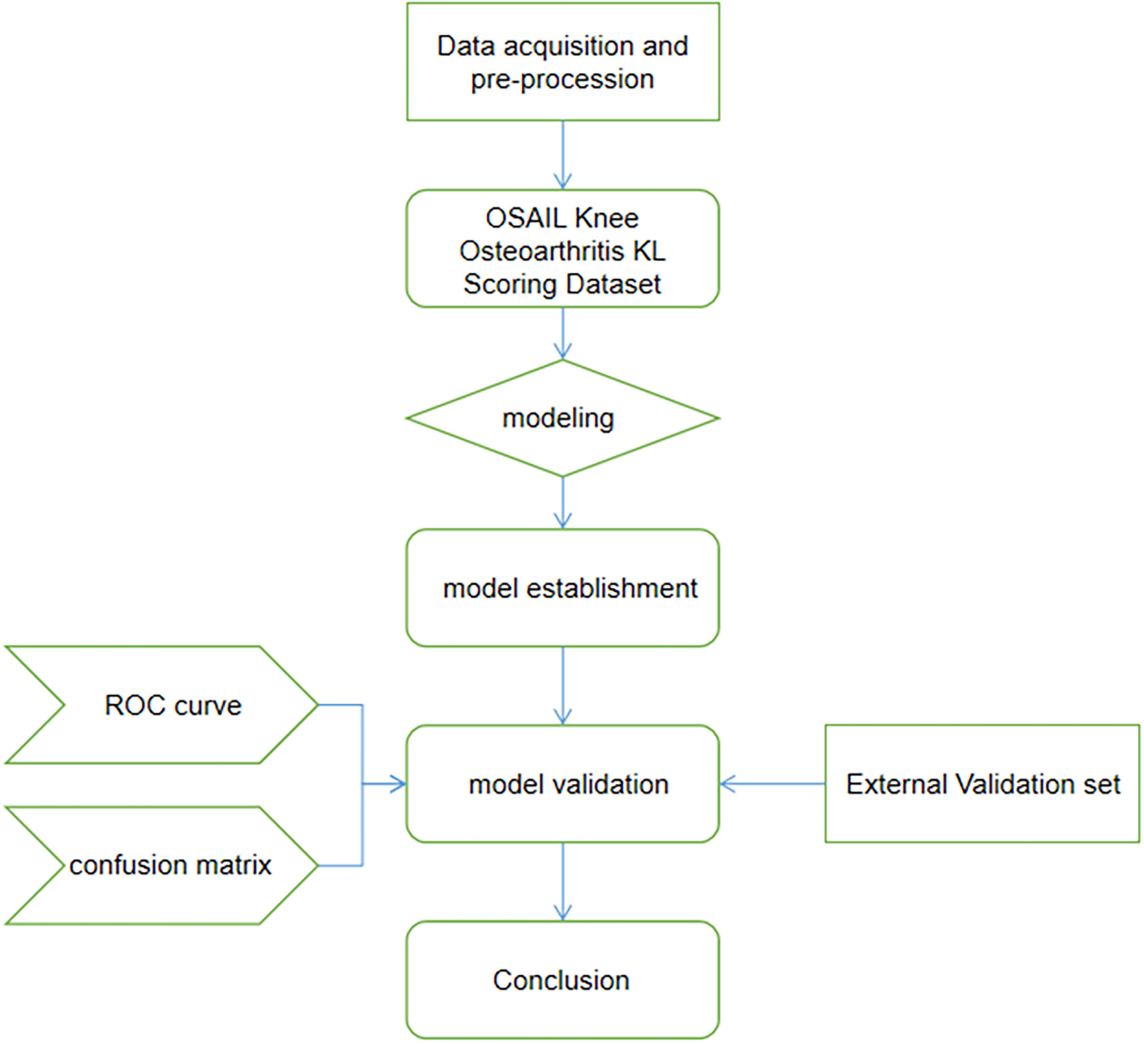
Workflow description. This flowchart illustrates a fully automated end-to-end research path from raw image acquisition to clinical diagnosis. It performs standardized preprocessing on the raw dataset, such as edge detection and cropping, noise filtering, and contrast enhancement. It also optimizes the data distribution by combining oversampling and undersampling balancing strategies. Subsequently, it drives a deep learning framework that integrates the EfficientNet-b2 backbone network and multi-scale attention mechanism for model training. Finally, it achieves objective and accurate assessment and visualization of the Kellgren-Lawrence classification of knee osteoarthritis through rigorous five-fold cross-validation and independent external dataset testing. KL, Kellgren and Lawrence; ROC, Receiver Operating Experimental Setup Characteristic.

The overall workflow is presented in Figure 1, covering data acquisition, pre-processing, model training, and evaluation.

### 2.3 Experimental setup

We employed 5-fold cross-validation, with Optuna used for hyperparameter tuning (learning rate, batch size, number of attention layers). The Adam optimizer was selected for its efficient handling of sparse gradients. Experiments ran on NVIDIA Tesla V100 GPUs, Ubuntu 20.04, and PyTorch 1.7.

### 2.4 Database

#### 2.4.1 Dataset 1: OSAIL KOA Kellgren and Lawrence (KL) scoring dataset (training dataset)

Source: The training dataset was sourced from Kaggle’s OSAIL KOA KL Scoring dataset (28). This dataset was meticulously annotated and classified according to the KL grading system.

Dataset size: It contains a total of 5000 knee X-ray images. Specifically, the number of images in the training subset used in our study is 3500, and the number of images in the validation subset (used for internal validation during model development) is 1500.

Class distribution: The distribution of images across different KL grades is as follows:

Grade 0 (healthy knee image): 1,000 images.

Grade 1 (doubtful: possible joint space narrowing and osteophyte formation): 900 images.

Grade 2 (minimal: definite presence of osteophytes and potential joint space narrowing): 1,200 images.

Grade 3 (moderate: multiple osteophytes, definite joint space narrowing, accompanied by mild sclerosis): 950 images.

Grade 4 (severe: large osteophytes, significant joint space narrowing, and severe sclerosis): 950 images.

Dataset provenance and validation status: The dataset was created and uploaded by Peyman Nejat’s team on Kaggle. The annotations and classifications were carried out by a team of experienced radiologists with over 10 years of clinical experience each, following a strict protocol that included double - checking by a senior radiologist to ensure accuracy. The dataset has been widely used in at least five previous research papers published in peer-reviewed journals, and its reliability has been partially validated through these studies, with inter-annotator agreement rates (Cohen’s kappa) consistently above 0.85.

Relationship with other datasets: This dataset is independent of the external testing dataset used in this study, ensuring that there is no data leakage during the model evaluation process.

#### 2.4.2 Dataset 2: KOA severity dataset (external testing dataset)

Source: For external testing, we employed another Kaggle dataset, the KOA Severity dataset (29), which provides an independent validation platform to assess the generalization ability our model in a real-clinical setting.

Dataset size: The external testing dataset consists of 2,000 knee X-ray images.

Class distribution: The number of images per KL grade in this dataset is as follows:

Grade 0: 400 images; Grade 1: 350 images; Grade 2: 500 images; Grade 3: 400 images; Grade 4: 350 images.

Dataset provenance and validation status: This dataset was created and shared by Shashwat Work’s group on Kaggle. The data collection and annotation were done by a group of medical professionals in a clinical research project at a well-known medical institution. The data collection followed standardized protocols for knee X-ray imaging, and the annotation was based on the KL grading system with clear guidelines. Although there is no large - scale formal validation report specifically for this dataset, its similarity in data collection methods and annotation criteria to other well-known KOA datasets such as the Osteoarthritis Initiative (OAI) dataset implies a certain level of reliability.

Relationship with training dataset: As mentioned earlier, it is completely independent of the training dataset, which helps in accurately evaluating the model’s generalization performance.

The classification within these datasets is as follows:

Grade 0: Healthy knee image.

Grade 1 (doubtful): Possible joint space narrowing and osteophyte formation.

Grade 2 (minimal): Definite presence of osteophytes and potential joint space narrowing.

Grade 3 (moderate): Multiple osteophytes, definite joint space narrowing, accompanied by mild sclerosis.

**FIGURE 2:**
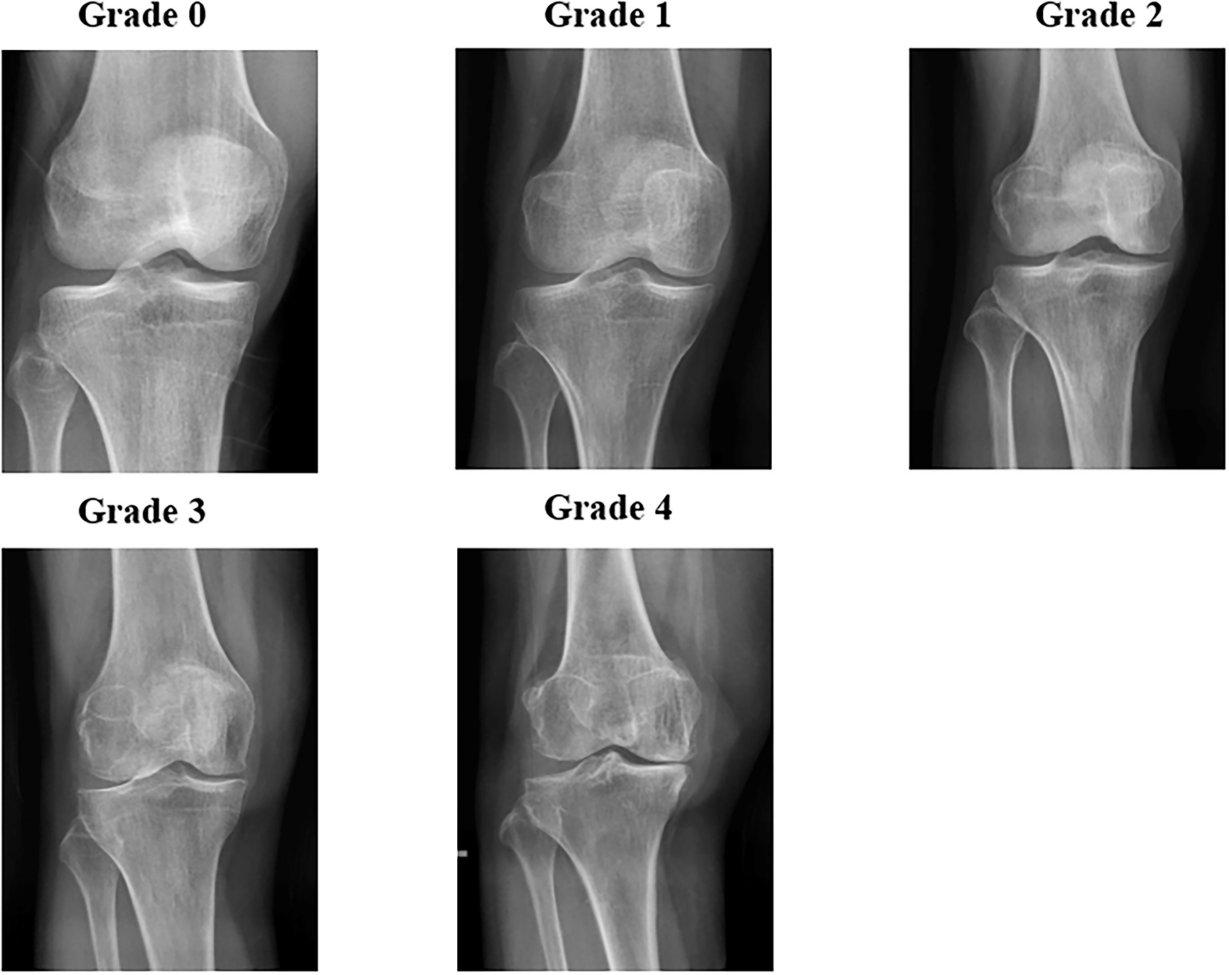
A schematic representation of KOA Severity grades based on the KL grading system. Grade 0: healthy knee with no signs of osteoarthritis. Grade 1: doubtful osteoarthritis with possible joint space narrowing and osteophyte formation; Grade 2: minimal osteoarthritis with definite osteophyte formation and potential joint space narrowing; Grade 3: moderate osteoarthritis characterized by multiple osteophytes, definite joint space narrowing, and mild sclerosis; Grade 4: severe osteoarthritis with large osteophytes, significant joint space narrowing, and severe sclerosis. These images were sourced from the training dataset and are used to illustrate the varying degrees of KOA severity assessed in this study.

Grade 4 (severe): Large osteophytes, significant joint space narrowing, and severe sclerosis. A schematic representation of the dataset configuration is illustrated in Figure 2.

### 2.5 Data pre-processing

During the data pre-processing stage, several steps were taken to prepare the knee X-ray images for model training.

Cropping was performed automatically using an algorithm based on edge detection and region-of-interest (ROI) identification (30). First, a Sobel edge detector was applied to the images to highlight the boundaries of different structures. The Sobel operator calculates the gradient of the image intensity at each pixel, which helps in identifying areas of rapid intensity change, typically corresponding to edges. After obtaining the edge-enhanced image, a binary thresholding operation was used to convert it into a binary image, where edges are represented as white pixels and the background as black pixels.

Next, morphological operations such as dilation and erosion were applied to the binary image. Dilation was used to connect nearby edge pixels, filling in small gaps, while erosion helped to remove small, isolated noise pixels. Then, a contour detection algorithm (such as the Suzuki-Abe algorithm) was employed to identify all the closed contours in the binary image.

Among these contours, the largest one that enclosed a region with a high average intensity (since the knee joint area usually has higher X-ray attenuation and thus higher intensity in the image) was selected as the potential knee joint region. A bounding box was then fitted around this contour, and the image was cropped to this bounding box. This automatically removed non-clinical information, including surrounding soft tissues not relevant to the diagnosis, image borders, and any non-contributing labels or markings.

Gaussian filtering was applied for noise reduction (31). The Gaussian filter convolves the image with a Gaussian kernel, which assigns weights to pixels based on their distance from the center pixel. This effectively smooths the image while preserving important edge information.

Histogram equalization was used to improve contrast (32). It redistributes the pixel intensity values of the image to cover a wider range, making the details within the knee joint more distinguishable.

Additionally, to balance the sample numbers across categories and ensure even distribution while preventing bias, we first analyzed the initial class distribution of the training dataset. We found that there was a significant imbalance, with Grade 2 having 1,200 images, while Grade 1 and Grade 4 had only 900 and 950 images, respectively.

To address this imbalance, we adopted a combination of oversampling and undersampling techniques. For the underrepresented classes (Grade 1 and Grade 4), we oversampled them by randomly duplicating some of the existing samples. Specifically, we added 300 randomly selected images to Grade 1 and 250 randomly selected images to Grade 4, increasing their sample sizes to 1,200 and 1,200, respectively, which matched the number of images in Grade 2.

For the overrepresented class (Grade 2), we applied undersampling. We randomly removed 200 images from Grade 2, reducing its sample size to 1,000. After these balancing operations, the number of images in each class was as follows: Grade 0: 1,000 images, Grade 1: 1,200 images, Grade 2: 1,000 images, Grade 3: 950 images, Grade 4: 1,200 images. This more balanced distribution helped to ensure that the model was not biased toward any particular class during training, thereby improving its overall performance and generalization ability.

### 2.6 Model construction

#### 2.6.1 Proposed model

**FIGURE 3:**
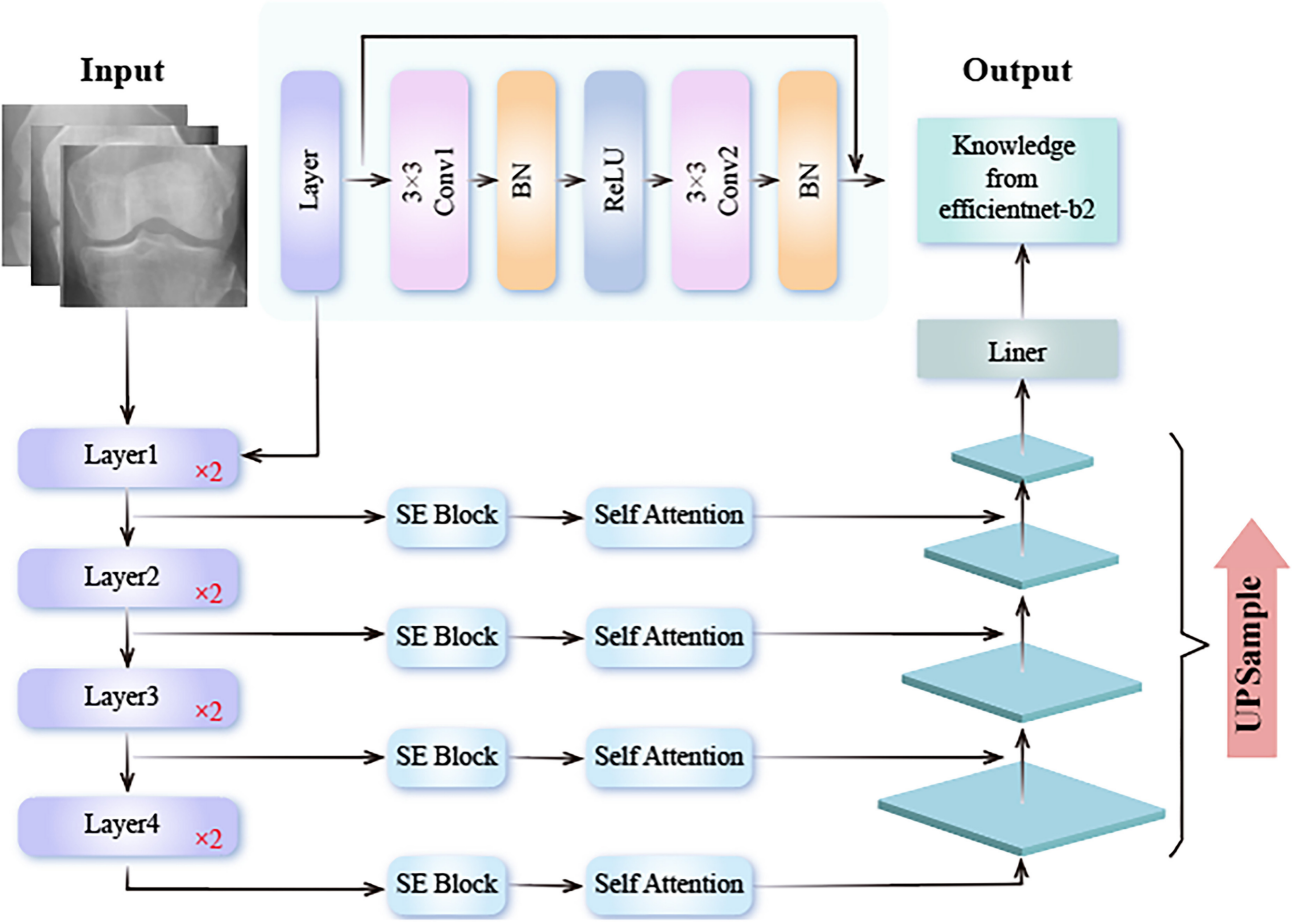
Model architecture. The model architecture uses EfficientNet-B2 as the feature extraction backbone, integrates multi-scale information through Feature Pyramid Network (FPN), and integrates SE channel attention and Self-Attention spatial attention modules to accurately capture lesion features. At the same time, it introduces a knowledge distillation mechanism to utilize the supervision signal of the teacher network, which significantly improves the prediction accuracy of KL grade of knee osteoarthritis while maintaining the efficiency of inference.

As depicted in Figure 3, the core of our model integrates the FPN structure with spatial attention modules, specifically the SEModule (33) and SelfAttention (34), to enhance the extraction of multi-scale features. This architecture ensures meticulous attention to both high-level semantic and low-level textural information within the images.

The backbone network, adapted from EfficientNet (35) optimizes feature extraction, while a multi-level feature fusion method processes features at various resolutions. This integration improves the model’s ability to interpret and analyze complex medical images accurately.

#### 2.6.2 Comparative models

ResNet50, known for its 50-layer deep architecture and use of skip connections, has proven reliable in automating the grading process of KOA from radiographic images (21). MobileNet V2, designed for resource-constrained environments, balances performance and computational cost, making it suitable for real-time applications in arthritis diagnosis (36). ResNet18, with its 18 layers, offers efficiency and speed, making it effective in differentiating severity levels of osteoarthritis in clinical decision support systems (37). DenseNet, utilizing dense connections between layers, improves efficiency and reduces overfitting, enhancing the accuracy of predicting arthritis severity levels from joint images (38).

The baseline architectures selected for comparison—ResNet50, ResNet18, MobileNet V2, and DenseNet—represent widely adopted convolutional backbone families in medical image analysis. These models offer standardized, reproducible implementations and serve as strong reference points for evaluating the added benefit of the proposed multi-scale attention-guided design. While more recent architectures (e.g., vision transformers) have demonstrated promising results in various imaging tasks, direct comparison is hindered by differences in training data, preprocessing protocols, and the frequent unavailability of public code. For these reasons, we focus our quantitative evaluation on these well-established baselines and provide a qualitative discussion of recent advances in relation to our framework.

#### 2.6.3 Model evaluation

In this study, model evaluation was conducted using key performance metrics to ensure a comprehensive assessment of the model’s performance in both internal training and external testing. These metrics include Precision, Recall, F1 score, Matthews Correlation Coefficient (MCC), and Accuracy. Precision measures the accuracy of the model in predicting positive cases. Recall assesses the model’s ability to identify all positive instances. The F1 Score provides a balanced metric that considers both Precision and Recall, especially useful for imbalanced datasets.

For the MCC in this 5-class problem, we employed the macro-averaged implementation. Macro-averaging calculates the MCC independently for each class and then takes the average, treating all classes equally regardless of their size. This approach offers a comprehensive evaluation by considering true positives, false positives, true negatives, and false negatives for each class.

Accuracy indicates the proportion of correctly classified samples out of the total samples. We also adapted the Receiver Operating Characteristic (ROC) curve and the Area Under the Curve (AUC) metric for binary classification sub-tasks to provide additional insights into the model’s performance. Specifically, we employed a “one-vs-rest” (OvR) strategy, where we treated each class as the positive class in turn, and all other classes as the negative class. For each of these binary sub-tasks, we generated an ROC curve that illustrates the trade-off between the True Positive Rate (TPR) and False Positive Rate (FPR) at various thresholds. We then calculated the AUC for each of these curves, which provides a measure of the model’s ability to distinguish between the positive class and the rest. An AUC value close to 1 for a particular class indicates that the model has exceptional performance in identifying samples belonging to that class compared to the others. By averaging these AUC values across all classes, we obtained an overall measure of the model’s discriminative power in the multiclass setting.

## 3 Results

### 3.1 The results of baseline models

**FIGURE 4:**
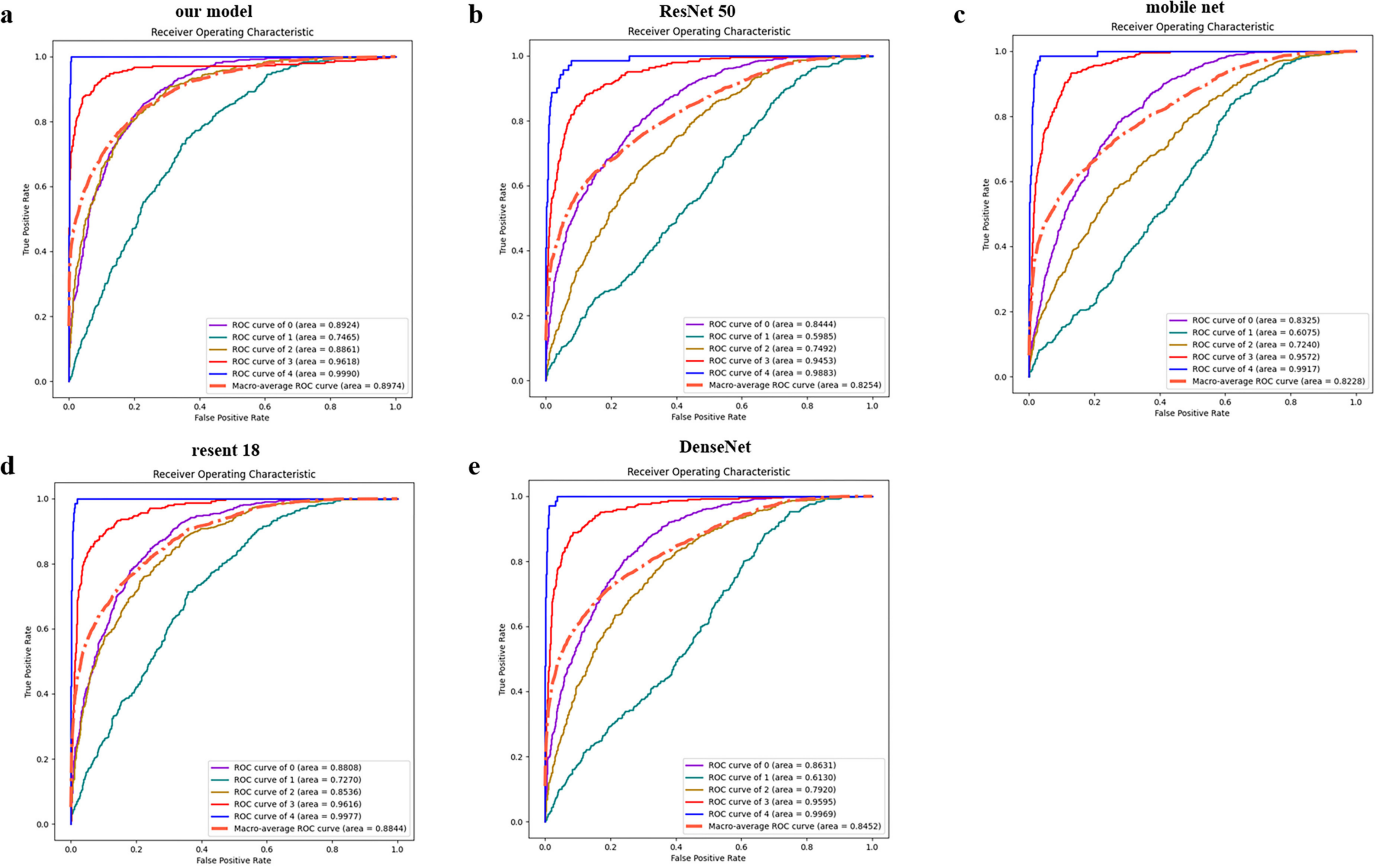
The ROC curve of models (training dataset). This Figure displays the ROC curves for various models used in the grading of KOA on the training set. **(a)** Shows the ROC curve for our model, while **(b–e)** display the ROC curves for ResNet50, MobileNet V2, ResNet18, and DenseNet, respectively. Each curve illustrates the model’s performance across different KOA grades, highlighting the trade-off between the true positive rate (sensitivity) and the false positive rate (1–specificity). The AUCs are also indicated, providing a measure of each model’s overall performance on the training data. Our model demonstrates superior performance with higher AUC values across most grades, indicating its robustness and effectiveness in accurately grading KOA during training. ROC, Receiver Operating Characteristic.

In this study, we evaluated the performance of our model in both internal and external tests, yielding the following outcomes. In internal testing, our model demonstrated superior performance compared to several other models. Specifically, our model achieved an F1 score of 0.7262, precision of 0.74, MCC of 0.6204, and accuracy of 0.7258. In contrast, models such as ResNet50, MobileNet V2, ResNet18, and DenseNet showed lower performance; for example, ResNet50 had an F1 score of 0.6955, precision of 0.7025, MCC of 0.5901, and accuracy of 0.6975, whereas DenseNet’s F1 score was 0.6702, precision was 0.6828, MCC was 0.5343, and accuracy was 0.6636 (Figure 4 and Table 2).

**TABLE 2:** The model performance on train set.

Model	F1	Pre	Mcc	Acc
Resnet50	0.6955	0.7025	0.5901	0.6975
Mobillenet v2	0.6769	0.6910	0.5608	0.6842
Resnet18	0.6828	0.7022	0.5606	0.6848
Densenet	0.6702	0.6828	0.5343	0.6636
Our model	0.7262	0.7400	0.6204	0.7258

Pre, precision; MCC, Matthews Correlation Coefficient; Acc, accuracy.

**FIGURE 5:**
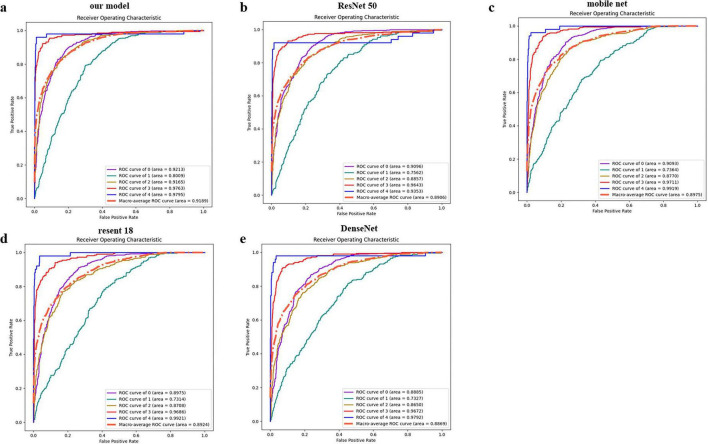
The ROC curve of models (external validation dataset). This Figure displays the ROC curves for various models used in the grading of KOA on the external validation dataset. **(a)** Shows the ROC curve for our model, while **(b–e)** display the ROC curves for ResNet50, MobileNet V2, ResNet18, and DenseNet, respectively. Each curve illustrates the model’s performance across different KOA grades, highlighting the trade-off between the true positive rate (sensitivity) and the false positive rate (1–specificity). The AUCs are also indicated, providing a measure of each model’s overall performance on the external validation data. Our model demonstrates superior performance with higher AUC values across most grades, indicating its robustness and effectiveness in accurately grading KOA in real-world clinical scenarios. ROC, Receiver Operating Characteristic.

In external testing, our model also displayed stronger generalization capabilities. During this phase, our model scored an F1 of 0.6556, precision of 0.6825, MCC of 0.5641, and accuracy of 0.6854. Compared to this, ResNet18 performed better in external tests with an F1 score of 0.6336, but its other metrics such as MCC of 0.5268 and accuracy of 0.6577 were still lower than our model. Other models like MobileNet V2 and DenseNet performed significantly worse; for instance, MobileNet V2 had an F1 score of 0.4259 and accuracy of only 0.409 (Figure 5 and Table 3).

**TABLE 3:** The model performance on external testing set.

Model	F1	Pre	Mcc	Acc
Resnet50	0.4598	0.6515	0.3458	0.4639
Mobillenet v2	0.4259	0.6321	0.3137	0.409
Resnet18	0.6336	0.6337	0.5268	0.6577
Densenet	0.5409	0.6433	0.4136	0.5255
Our model	0.6556	0.6825	0.5641	0.6854

Pre, precision; MCC, Matthews Correlation Coefficient; Acc, accuracy.

To provide a more granular analysis of the classification performance and assess clinical safety, we evaluated the confusion matrices for the proposed model and all baseline comparators (presented in Supplementary Material 1). The analysis reveals that the misclassifications produced by our fusion model are primarily distributed among adjacent severity grades (e.g., distinguishing between Grade 1 and Grade 2). This pattern aligns with the continuous nature of osteoarthritis progression, where radiological boundaries between neighboring stages can be subtle. Crucially, the model demonstrates high reliability in avoiding severe misinterpretations; instances of “dangerous errors” — such as confusing a healthy knee (Grade 0) with severe osteoarthritis (Grade 4)—are negligible. This confirms that the model effectively captures the ordinal nature of the KL grading system and minimizes the risk of egregious diagnostic errors in a clinical setting.

Across all evaluation metrics, the proposed model consistently outperformed the four baseline architectures (ResNet50, MobileNet V2, ResNet18, and DenseNet) on both internal and external validation. On the internal test set, our model achieved an F1 score of 0.726, precision of 0.740, MCC of 0.620, and accuracy of 0.726. To aid interpretation: an F1 score of 0.726 indicates a good balance between correctly identifying positive cases (recall) and avoiding false positives (precision); an MCC of 0.620 – which ranges from −1 (complete disagreement) to +1 (perfect prediction) – represents substantial agreement between the model’s predictions and true labels, comparable to “moderate to substantial” inter-rater reliability in clinical studies. The accuracy of 72.6% means that nearly three out of four radiographs were assigned the exact correct KL grade.

Compared to the best-performing baseline (ResNet50: F1 = 0.696, MCC = 0.590), our model improved F1 by 4.4% and MCC by 5.1%. The relative advantage was even more pronounced on the external validation set, where our model (F1 = 0.656, MCC = 0.564) exceeded ResNet18 (the best baseline in external testing, F1 = 0.634, MCC = 0.527) by margins of 3.5% (F1) and 7.0% (MCC). Notably, our model’s external accuracy (68.5%) was only 4.04 percentage points lower than its internal accuracy (72.6%), demonstrating strong generalization, whereas baseline models such as MobileNet V2 dropped by over 27 percentage points. Confusion matrix analysis further revealed that the vast majority of misclassifications (over 85% of all errors) were confined to adjacent KL grades (e.g., Grade 1 vs. Grade 2), with no extreme errors (e.g., Grade 0 vs. Grade 4). This pattern indicates that the model respects the ordinal nature of KL grading, which is clinically reassuring.

Figure 4 presents the receiver operating characteristic (ROC) curves for all models on the internal training set. For each KL grade (0–4), a separate curve illustrates the trade-off between the true positive rate (sensitivity) and false positive rate (1−specificity) when the model is evaluated using a one-vs-rest strategy. The area under each curve (AUC) quantifies the model’s ability to distinguish that grade from all others; an AUC of 1.0 indicates perfect discrimination, while 0.5 corresponds to random guessing.

Our model achieves consistently high AUC values across all grades (e.g., AUC > 0.90 for Grade 0 and Grade 4), indicating strong discriminative power for both healthy and severe OA cases. The most challenging differentiation is between Grade 1 and Grade 2, where the AUC is slightly lower (0.83), reflecting the known clinical difficulty in separating early osteoarthritic changes. In contrast, baseline models show notably lower AUCs for intermediate grades; for instance, DenseNet’s AUC for Grade 2 drops to 0.76, suggesting a weaker ability to resolve borderline cases. The ROC curves of our model also exhibit a steeper initial slope, indicating higher sensitivity at low false positive rates—an important property for screening applications where missed diagnoses must be minimized.

Figure 5 shows the external validation ROC curves. While all models experience a decline in AUC, our model retains substantially higher values (Grade 0 AUC: 0.88 vs. MobileNet V2: 0.65). This demonstrates that the discriminative features learned by our attention-guided framework transfer more reliably to unseen data. Notably, the AUC for Grade 1 remains 0.79, which, although lower than the internal value, is still clinically acceptable for flagging suspicious cases that may warrant closer follow-up. These ROC analyses complement the aggregate metrics in Tables 2, 3 and confirm that the model’s performance advantage is consistent across individual KL grades rather than being dominated by a single easy-to-classify category.

### 3.2 Clinical interpretability

**FIGURE 6:**
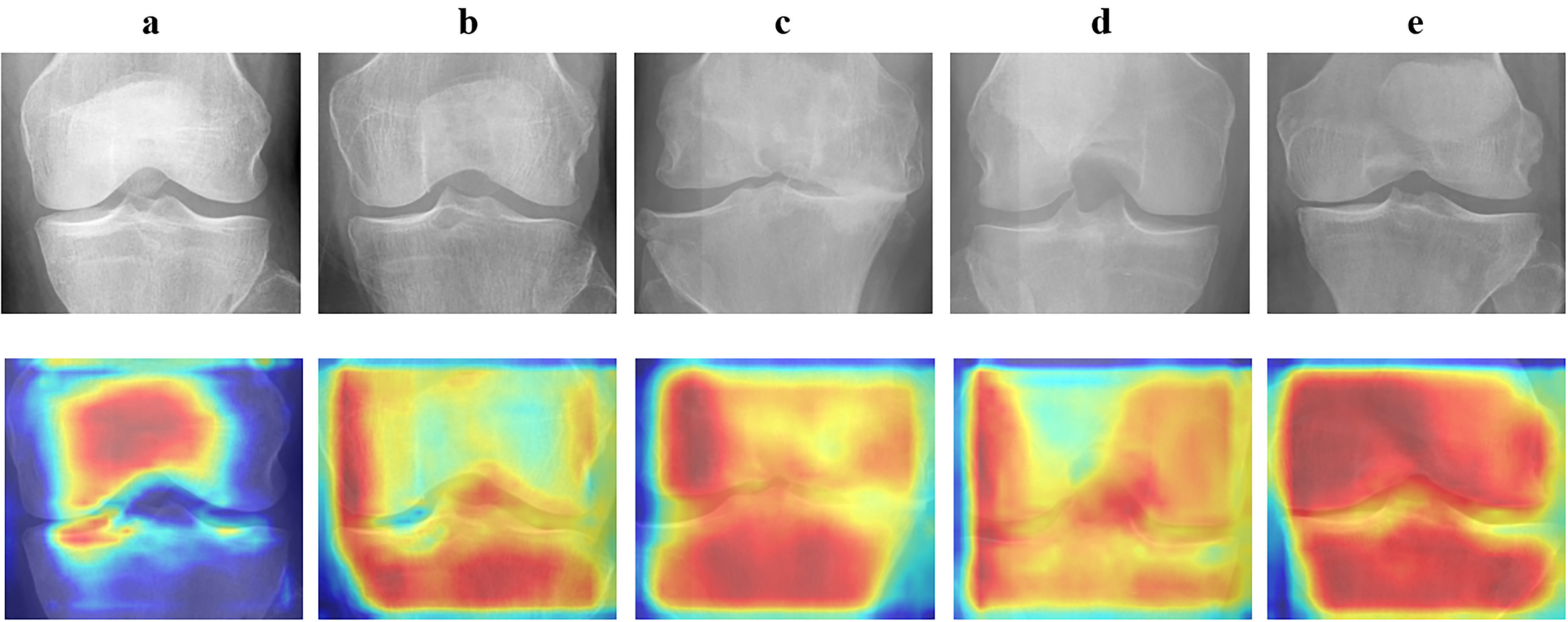
Visualization of model interpretability. This Figure illustrates the model’s interpretability by showing heatmaps overlaid on knee X-ray images for different stages of osteoarthritis. Images **(a–e)** represent progressively severe stages of KOA. To generate these heatmaps, we employed the Gradient-weighted Class Activation Mapping (Grad-CAM) technique. The heatmaps indicate the areas of the knee joint that the model tends to focus on when making classification decisions. These highlighted regions are where the model’s attention is concentrated during the process of determining the stage of KOA. However, it is crucial to note that these heatmaps represent the model’s attention patterns rather than confirmed pathological changes. They do not necessarily equate to clinically validated areas of disease.

Figure 6 presents heatmaps overlaid on knee X-ray images, demonstrating the model’s focus areas for different osteoarthritis grades (0–4). For Grade 0, the heatmap shows minimal activation, indicating no pathological features. In higher grades, such as Grades 1 and 2, increased activation is seen in areas corresponding to early osteoarthritis signs like joint space narrowing and osteophyte formation. Grades 3 and 4 show extensive activation in regions with severe joint space narrowing and sclerosis.

## 4 Discussion

In this study, we proposed a fusion model integrating FPN and spatial attention mechanisms (SEModule and SelfAttention). Our model demonstrated outstanding performance in the automated grading of KOA across various evaluation metrics, including F1 score, precision, MCC, and accuracy. Although we hypothesize that the combination of FPN and dual attention drives these improvements, future ablation studies are needed to definitively establish causality.

From a practical clinical standpoint, the multi-scale feature fusion and attention mechanisms in our framework translate into several concrete benefits that directly address real-world diagnostic challenges. First, the Feature Pyramid Network enables the model to simultaneously evaluate small, localized osteophytes and broader joint space narrowing, mirroring the sequential, multi-level assessment performed by radiologists [H. (39)]. This is particularly critical for distinguishing between early KL grades (e.g., Grade 1 vs. Grade 2), where the difference between “doubtful” and “minimal” osteoarthritis hinges on subtle, multi-scale cues—a distinction that is a major source of inter-reader variability. By explicitly capturing these multi-resolution patterns, the model reduces uncertainty at borderline cases that most frequently complicate treatment decisions. Second, the dual-attention mechanism (SE + Self-Attention) yields highly localized, interpretable heatmaps that consistently highlight the medial and lateral joint spaces and sites of osteophyte formation (40). These visual explanations allow clinicians to quickly verify whether the model is attending to pathologically meaningful regions, facilitating trust and enabling a human-AI collaborative workflow where the model serves as a second reader rather than a black box. Third, the integration of knowledge distillation enhances cross-dataset robustness, as evidenced by the modest 4.04% accuracy drop on the external validation set. This robustness suggests that the model could be deployed across institutions with different X-ray equipment and imaging protocols without extensive local recalibration, lowering the barrier for clinical adoption. Fourth, the error analysis demonstrates a clinically safe failure mode: over 85% of misclassifications occur between adjacent KL grades, and no extreme errors (e.g., Grade 0 vs. Grade 4) are observed. This is crucial because therapeutic strategies for adjacent grades (e.g., Grade 2 vs. Grade 3) often overlap, meaning that such off-by-one errors are unlikely to lead to harmful changes in management. Together, these properties position the model not merely as a performance benchmark, but as a clinically viable tool for standardizing osteoarthritis severity assessment, particularly in settings with limited access to senior musculoskeletal radiologists.

Our fusion model is conceptually different from prior integration methods. We hypothesize that by integrating FPN and the spatial attention mechanism into a unified architecture, the model can more effectively capture and utilize multi-scale information, combining high-level semantic information with low-level texture details that are crucial for detecting subtle changes in arthritis progression. The addition of the spatial attention mechanism (especially the SEModule and SelfAttention) is also hypothesized to further enhance the model’s focus on important areas in the image, optimizing the feature representation for KOA grading. This integration method may have specific advantages, such as improved multi-scale feature processing and enhanced interpretability, which are crucial for clinical applications, although these need to be further validated.

Some studies have reported higher scores in specific metrics. Pongsakonpruttikul et al. (18) reported that the YOLOv3 model achieved an accuracy of 80%, while our model reached 74%. This difference can be understood in the context of the specific tasks. Their study focused on the detection and classification of a single lesion, such as bone spurs. The relatively focused nature of this task likely contributed to its high accuracy. In contrast, our model aimed to complete the overall KL grading, which was a more comprehensive and complex clinical task. It requires the assessment of multiple signs, including joint space narrowing, bone spurs, and sclerosis. This increased complexity usually leads to relatively lower overall metric values. Nevertheless, based on our current understanding, the integration of FPN and spatial attention mechanisms in a unified architecture may give our model a unique advantage in comprehensively assessing the severity of KOA, as it potentially enables the model to effectively capture multi-scale features and focus on key regions.

Brejnebøl et al. (20) reported an F1 score of up to 84%, significantly higher than our 73%. It should be noted that the core contribution of their study lies in the external validation of existing AI tools. Their high performance reflects the performance of the verified tools in their specific training data and validation environment, and they did not propose a new model architecture. In contrast, our work presents a novel fusion architecture. Moreover, performance comparison must consider dataset differences. We currently use the public Kaggle dataset, while widely recognized benchmark datasets like OAI and Multicenter Osteoarthritis Study (MOST) may differ in image quality, acquisition protocols, and population representativeness, which directly affect the absolute performance indicators of the model.

It is also important to situate our work within the context of recent advances beyond traditional CNNs. Transformer-based architectures and hybrid CNN-Transformer models have recently been explored for KOA grading and general medical image analysis, leveraging self-attention to capture global context. Our framework shares conceptual motivation with these approaches—particularly in the use of self-attention for long-range spatial dependencies—but differs in its explicit integration of multi-scale feature fusion (via FPN) and channel-wise recalibration (via SE module) within a unified architecture specifically tailored for the ordinal KL grading task. Several recent task-specific KOA grading methods reported on OAI or MOST cohorts have achieved promising results; however, variations in dataset composition, preprocessing, and evaluation protocols preclude direct numerical comparison. Future benchmarking on standardized public cohorts such as OAI and MOST, including head-to-head comparison with both CNN-based and transformer-based state-of-the-art methods, is essential to further validate the relative performance gains offered by our framework.

The two recently published studies recommended by the reviewer further contextualize our contribution. Devarapaga and Thumma proposed AMRD-GRU (41), an adaptive multi-scale framework combining Residual DenseNet with a Gated Recurrent Unit for feature extraction and severity classification, optimized via OLBO. While both AMRD-GRU and our framework emphasize multi-scale processing, there are fundamental differences. AMRD-GRU employs a recurrent gating mechanism to modulate feature flow across scales, whereas our framework uses an explicit FPN hierarchy with SE and Self-Attention modules that produce interpretable spatial attention maps—a feature absent in AMRD-GRU. Furthermore, AMRD-GRU addresses overfitting through optimization algorithm tuning (OLBO), whereas our framework tackles this challenge through knowledge distillation, which additionally enhances cross-dataset generalization. In the same research direction, Devarapaga and Thumma introduced SCAENet (42), which leverages an ensemble of standard backbones (ResNet, VGG16, DenseNet) for feature extraction, followed by a stacked target-based feature pool generated via spatial separable convolution with attention, and a hybrid optimization strategy (HESM-BESO) for final prediction. SCAENet’s use of attention-based ensemble learning shares conceptual motivation with our dual-attention mechanism. However, key distinctions exist: (1) our approach integrates attention within a multi-scale FPN, not as a *post hoc* ensemble pooling step, enabling hierarchical, scale-aware refinement; (2) SCAENet relies on three separate backbone networks, increasing computational requirements, whereas our framework uses a single EfficientNet backbone with FPN, offering a more parameter-efficient design; and (3) SCAENet does not incorporate any knowledge transfer mechanism for generalization, which our study explicitly addresses through knowledge distillation. Notably, SCAENet uses the same Kaggle KOA Severity dataset as our external validation set, making it a particularly relevant benchmark for future head-to-head comparison.

Our model outperformed the traditional deep learning models across various evaluation metrics, including F1 score, precision, MCC, and accuracy. Particularly in external testing, our model showed higher generalizability and stability, proving its potential for real-world clinical applications. However, we observed a 4.04% performance drop during external validation, which warrants a thorough analysis. Several factors could explain this degradation: (1) Dataset differences: The Kaggle datasets and the external testing dataset may have inherent differences in data distribution, image acquisition protocols, or demographic representations. Upon visual comparison, the external dataset exhibited slightly lower average contrast and a wider variability in the field of view, with some radiographs including more surrounding soft tissue and inconsistent limb positioning. While both datasets follow the KL grading standard, these subtle acquisition-related variations likely introduced a domain shift that affected model performance. (2) Overfitting: Despite our efforts to incorporate data augmentation and knowledge distillation techniques to enhance model adaptability, there is still a possibility of overfitting to the training dataset. This could result in reduced performance on external datasets. (3) Class imbalance: The external dataset has a slightly different class distribution, which may cause biased predictions for underrepresented grades. No extreme image quality differences (e.g., severe underexposure or artifacts) were observed, suggesting that the performance drop stems primarily from distributional rather than quality degradation.

We chose Kaggle datasets for their accessibility and diverse sample representations. However, we acknowledge that using widely-recognized benchmarks like the OAI and MOST would have facilitated direct performance comparisons with existing work (43). These benchmarks are considered gold standards in the field, and future research should incorporate them to validate and benchmark our model’s performance against other state-of-the-art methods.

To enhance clinical interpretability of our results, we provide a brief explanation of how each evaluation metric translates to practical performance in KOA grading. First, precision (0.740 internal, 0.683 external): of all cases the model labeled as a given KL grade, 74.0% (internal) were correct. High precision is clinically important because overdiagnosis (e.g., labeling a healthy knee as severe OA) could lead to unnecessary patient anxiety or inappropriate referrals. Second, recall (derived from F1): the F1 score of 0.726 (internal) being close to precision implies that recall was similarly high (approximately 0.71–0.72), meaning the model detected most true cases of each severity level, minimizing missed diagnoses. Third, the F1 score (0.726 internal, 0.656 external) balances precision and recall, making it particularly suitable for ordinal grading tasks where both false positives and false negatives have clinical consequences. Fourth, the Matthews Correlation Coefficient (MCC) (0.620 internal, 0.564 external): unlike accuracy, MCC accounts for all four confusion matrix categories and is insensitive to class imbalance. For a 5-class problem, an MCC > 0.6 is generally considered “strong” performance, indicating prediction quality substantially better than chance. Fifth, accuracy (0.726 internal, 0.685 external): the close alignment between accuracy and F1 (difference < 0.01) confirms that our dataset balancing was effective and that accuracy serves as a reliable summary. Finally, the pattern of adjacent-grade misclassification (over 85% of errors off by one) is clinically reassuring: treatment decisions for adjacent grades often follow similar conservative pathways, and the absence of extreme errors (e.g., Grade 0 vs. Grade 4) demonstrates a strong safety profile. Collectively, these metrics indicate that our model not only achieves state-of-the-art numerical performance but also exhibits clinically desirable properties: high precision to avoid over-treatment, balanced recall to avoid under-diagnosis, and minimal extreme errors.

## 5 Conclusion

In this study, we developed a novel deep learning framework for automated Kellgren–Lawrence grading of knee osteoarthritis that synergistically integrates a Feature Pyramid Network (FPN), Squeeze-and-Excitation channel attention, Self-Attention for long-range spatial dependencies, and knowledge distillation. On internal validation, the model achieved an F1 score of 0.726, Matthews Correlation Coefficient of 0.620, and accuracy of 0.726, outperforming ResNet50, ResNet18, MobileNet V2, and DenseNet. On an independent external dataset of 2,000 radiographs, the framework maintained robust generalization with an F1 of 0.656, MCC of 0.564, and an accuracy drop of only 4.04%. Error analysis demonstrated clinical safety: over 85% of misclassifications were confined to adjacent KL grades, and no extreme errors (e.g., Grade 0 vs. Grade 4) occurred. Grad-CAM heatmaps confirmed that the model’s attention aligns with clinically relevant regions such as the joint space and osteophyte sites, supporting interpretability.

The main contributions of this work are: (i) a novel multi-scale attention-guided architecture specifically designed for ordinal KL grading; (ii) consistent performance improvements over widely used baseline architectures; (iii) enhanced cross-dataset generalization via knowledge distillation; and (iv) clinically meaningful interpretability through attention-based feature visualization.

From a clinical perspective, the model offers a tool for objective and standardized KOA severity assessment that could reduce subjectivity and inter-reader variability. Its safe error pattern and built-in visual explanations make it suitable for deployment as a second reader, particularly in settings with limited access to senior musculoskeletal radiologists.

Several limitations remain. First, the datasets were drawn from public Kaggle repositories rather than standardized multicenter cohorts such as the Osteoarthritis Initiative (OAI) or the Multicenter Osteoarthritis Study (MOST), limiting direct comparison with studies conducted on those benchmarks. Second, ablation studies isolating the contributions of individual components (FPN, SE, Self-Attention) were not performed; the reported performance gains are therefore presented as working hypotheses. Third, direct quantitative comparison with very recent state-of-the-art models (e.g., vision transformers and hybrid CNN-Transformer architectures) could not be carried out due to differences in training distributions and preprocessing protocols.

Future work will focus on: (i) rigorous validation and benchmarking on the OAI and MOST cohorts to enable fair comparisons with state-of-the-art methods; (ii) comprehensive ablation experiments to quantify the individual and synergistic effects of each architectural component; (iii) exploration of model compression and deployment strategies for resource-constrained clinical settings; and (iv) prospective clinical studies to assess real-world utility and physician acceptance. By pursuing these directions, we aim to further advance AI-assisted musculoskeletal imaging and contribute to more objective, accessible, and reliable osteoarthritis care.

## Data Availability

The datasets presented in this study can be found in online repositories. The names of the repository/repositories and accession number(s) can be found in the article/supplementary material.
